# Psychiatric Morbidities and Their Impact on Quality of Life in Patients With Psoriasis

**DOI:** 10.7759/cureus.43394

**Published:** 2023-08-12

**Authors:** Abhishek Kumar, Dhrubajyoti Bhuyan, Shyamanta Barua, Sabita Dihingia, Lakshimi Borgohain

**Affiliations:** 1 Psychiatry, Diphu Medical College and Hospital, Diphu, IND; 2 Psychiatry, Assam Medical College and Hospital, Dibrugarh, IND; 3 Dermatology, Assam Medical College and Hospital, Dibrugarh, IND; 4 Psychiatry, Nalbari Medical College and Hospital, Nalbari, IND; 5 Psychiatry, Lakhimpur Medical College and Hospital, Lakhimpur, IND

**Keywords:** quality of life, psychiatric morbidities, anxiety, depression, skin

## Abstract

Background

Psychiatric morbidities, which are commonly seen in psoriasis patients, are often missed or ignored leading to poor quality of life. A lack of communication between psychiatrists and dermatologists leads to ignorance of psychiatric morbidities in psoriasis patients, which can eventually take a severe form. Therefore, both groups must collaborate to provide high-quality patient care. As there is a dearth of such studies in the North-Eastern part of India, this study aimed to highlight the above-mentioned problem.

Aim

The aim of this study was to study psychiatric morbidities in patients with psoriasis and to compare quality of life in psoriasis patients with and without psychiatric morbidities.

Methods

This study was a hospital-based cross-sectional study conducted in the Dermatology Department, Assam Medical College and Hospital, Dibrugarh, Assam, India from July 2020 to July 2021. Ninety patients with psoriasis were included in the study and the diagnosis was confirmed by a consultant dermatologist, then the Mini International Neuro-psychiatric Interview Scale (M.I.N.I.; version 5.0) scale was applied to screen psychiatric morbidities. The diagnoses were confirmed using ICD-10 followed by dividing psoriasis patients into two groups, i.e. with and without psychiatric morbidities. After that the World Health Organization Quality of Life (WHOQOL) scale was applied to both groups and the domains of quality of life were compared.

Results

Our results showed that 61.1% of psoriatic patients had psychiatric morbidities, which is abnormally high compared to other dermatological disorders. The psychological domain of quality of life was the most affected (WHOQOL scale scoring of 38.12±6.67 vs. 48.76±6.21) in both groups of patients (i.e., with and without psychiatric morbidities), and the environmental domain was the least affected (56.67±10.65 vs. 64.67±8.18). Every domain of quality of life had a lower score in patients with psoriasis with psychiatric morbidities as compared to those without (*p*<0.05).

Conclusion

Our results of 61.1% psychiatric morbidities in psoriasis patients emphasize the need for psychiatric evaluation in every psoriasis patient. The timely intervention of psychiatric morbidity in psoriasis patients with collaboration of psychiatrists and dermatologists will surely improve the patient’s condition to some extent and, thus, their quality of life.

## Introduction

Psoriasis is a dermatological disorder with a complex relationship between skin and mind. Brain, nerve, and skin are embryological derivatives of the ectoderm, and, to be more specific, are derived from the neural plate, thus forming their association [1]. Approximately 30%-40% of patients seeking medical treatment for skin conditions have an underlying psychological problem or psychiatric illness that causes or exacerbates the skin disorder [2]. In psoriasis, the prevalence of psychiatric disorders is significantly much higher than in the general population [3]. Mood symptoms have been shown to be more prevalent in skin disorders involving disfigurement [4]. Different studies have found that the area of skin affected can influence the patient’s quality of life. Furthermore, the more severe the symptoms, the more likely patients have to take working days off and suffer reduced productivity. Psoriasis may also disrupt daily activities due to the itching of lesions and the burning sensation [5]. Numerous studies have proven that psychiatric disorders can result from psoriasis, which have a potential effect on mental health and, in turn, a worsening effect on overall quality of life. In light of the growing number of patients with psoriasis in the outpatient department of dermatology in this geographical region of our country, this study was undertaken with the aim to assess their psychiatric morbidities and quality of life.

This paper was previously presented as a free paper at the Annual National Conference of Indian Psychiatric Society 2022 held from 24th to 26th March, 2022.

## Materials and methods

**Figure 1:**
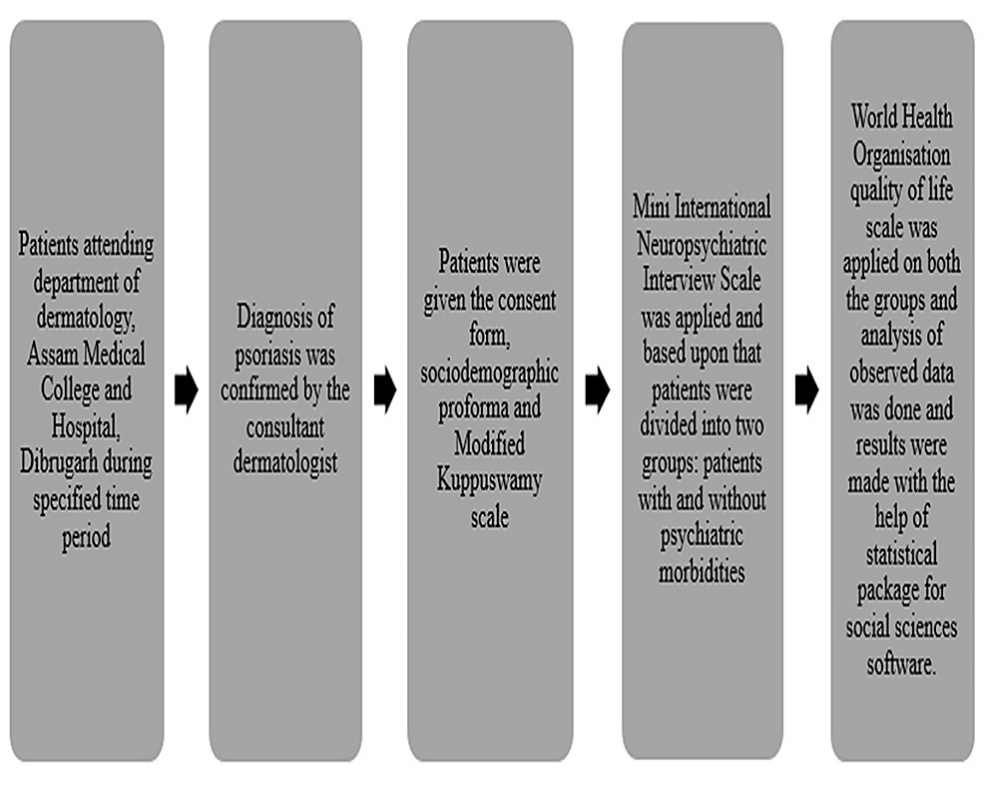
Schematic representation of the study

The study was conducted in the Department of Dermatology and the Department of Psychiatry, Assam Medical College, Dibrugarh, Assam, India. It was a cross-sectional study conducted over a period of one year. The study protocol was approved by the hospital’s institutional ethics committee (approval AMC/EC/PG/8838). Psoriasis patients aged 18-60 years attending the hospital’s dermatology outpatient department were considered and selected randomly in the period of time of study (Figure 1). Those suffering from any substance dependence, pre-existing psychiatric illnesses, or co-morbid medical illness likely to cause psychiatric morbidities such as diabetes mellitus, hypertension, cardiovascular disease, or thyroid dysfunction were excluded from the study. A total of 90 patients were included in the study after obtaining written informed consent from them. A structured proforma was used to record the sociodemographic data. The sociodemographic data, the modified Kuppuswamy Scale updated for the year 2019 [6] were applied and later the Mini International Neuro-psychiatric Interview scale (M.I.N.I.; version 5.0) [7] was used to screen psychiatric morbidity in the psoriasis patients, and the ICD-10 Criteria were used to confirm the diagnosis. The M.I.N.I. is a short structured clinical interview that allows researchers to diagnose psychiatric disorders according to DSM-IV or ICD-10. The time for administration of the interview is around 15 minutes and was mainly used for epidemiological studies and multicenter clinical trials. After that patients were divided into two groups i.e. patients of psoriasis with and without psychiatric morbidities. Then, the World Health Organization Quality of Life (WHOQOL) scale was applied to both groups and they were compared for their quality of life. Individual's perceptions of their position in life assessed by the WHOQOL scale in the context of the culture and value systems in which they reside and in relation to their aims, expectations, concerns and standards. It was evolved collaboratively in some 15 cultural settings over a number of years and has now been field tested in 37 field centers. It is a 100-question assessment that presently exists in directly comparable forms in 29 language versions. It gives a multi-dimensional profile of scores across domains and sub-domains (facets) of quality of life. Finally, a statistical analysis of the data was performed with the Statistical Package for Social Sciences (SPSS version 16; Chicago, IL, USA) after the end of the study.

Statistical analysis

Continuous data are presented as means ± standard deviations, and categorical data are presented as frequencies (%). Statistical significance was assessed using Student’s t-test [8] for continuous data and the chi-squared test [9] or Fisher’s exact test [10] for categorical data, where a p-value of <0.05 was considered statistically significant. Finally data of each scale were collected and statistical analysis were done using SPSS version 16.0.

## Results

Table 1 shows that out of the 90 patients included in study, the 31-40-years age group was the largest (n=35, 38.9%). The majority of patients were male (n=56, 62.2%), from an urban background (62.2%), had a primary school certificate educational level (25.6%), and belonged to the upper lower socioeconomic class (n=47, 52.2%), as per the Kuppuswamy Socioeconomic Scale updated for version 2019. The numbers of joint and nuclear families were equal (n=45, 50%) in the study population. The majority of the population in the study group were married (n=70, 77.8%). Table 2 lists the prevalence of psychiatric morbidities in the psoriasis patients. It was evident from the data that 55 out of 90 patients (61.1%) had psychiatric morbidities.

**Table 1:** Sociodemographic distribution of the study population.

Variables	Number(n)	Percentage(%)
AGE GROUP		
≤20	5	5.6
21-30	26	28.9
31-40	35	38.9
41-50	20	22.2
>50	4	4.4
SEX		
Male	56	62.2
Female	34	37.8
LOCALITY		
Rural	34	37.8
Urban	56	62.2
FAMILY TYPE		
Joint	45	50.0
Nuclear	45	50.0
EDUCATION		
Graduate or post-graduate	18	20.0
High School Certificate	18	20.0
Middle School Certificate	15	16.7
Primary School Certificate	23	25.6
Illiterate	16	17.8
SOCIOECONOMIC STATUS		
Upper Middle (II)	8	8.9
Lower Middle (III)	33	36.7
Upper Lower (IV)	47	52.2
Lower (V)	2	2.2
MARITAL STATUS		
Married	70	77.8
Single/Widow/Widower/Divorced	20	22.2

**Table 2:** Prevalence of psychiatric morbidities in patients with psoriasis.

Patients With Psoriasis	Number(n)	Percentage(%)
With psychiatric morbidities	55	61.1
Without psychiatric morbidities	35	38.9

Table 3 lists the percentages of different psychiatric morbidities in the psoriasis patients. The most common psychiatric illness associated with psoriasis was depressive episode (n=30, 54.5%), followed by anxiety disorder, unspecified (n=13, 23.6%), social phobia (n=5, 9.09%), panic disorder (n=3, 5.45%), and obsessive-compulsive disorder (n=4, 7.27%). None of the patients had unspecified psychosis not due to a substance or known physiological condition as a psychiatric morbidity.

**Table 3:** Psychiatric morbidities in psoriasis patients.

Psychiatric Illness Associated With Psoriasis	Number(n)	Percentage(%)
Depressive episode	30	54.5
Anxiety disorder, unspecified	13	23.6
Social phobia	5	9.09
Unspecified psychosis not due to a substance or known physiological condition	0	0.00
Panic disorder (episodic paroxysmal anxiety)	3	5.45
Obsessive-compulsive disorder	4	7.27

Table 4 shows comparison between patients with psoriasis with and without psychiatric morbidities. However, none of these associations were statistically significant.

**Table 4:** Comparison of psychiatric morbidities in the study population.

Variables	With Psychiatric Morbidities(n=55)			Without Psychiatric Morbidities(n=35)		p-value
	Number		Percentage	Number	Percentage	
Age Group						
≤20	2	40.0		3	60.0	0.23
21-30	24	92.4		2	7.6	
31-40	15	44.2		19	55.8	
41-50	14	66.6		7	33.4	
>50	0	0.0		4	100	
Gender						
Male	32	57.1		24	42.9	0.06
Female	23	67.6		11	32.4	
Domicile						
Rural	22	64.8		12	35.2	0.06
Urban	33	58.9		23	41.1	
Education of head of family						
						0.09
Graduate	8	44.4		10	55.6	
High school certificate	8	44.4		10	55.6	
Middle school certificate	10	66.7		5	33.3	
Primary school certificate	16	69.6		7	30.4	
Illiterate	14	87.5		2	12.5	
Socioeconomic status						
						0.14
II	5	62.5		3	37.5	
III	18	54.5		15	45.5	
IV	32	68.1		15	31.9	
V	0	0.0		2	100	
Type of family						
Nuclear	22	48.9		23	51.1	0.22
Joint	33	73.3		12	26.7	
Marital status						
Unmarried	8	40		12	60	0.23
Married	47	67.1		23	32.9	
Separated	0	0.0		0	100	

Table 5 shows that every domain of quality of life was more affected in psoriasis patients with psychiatric morbidities as compared to those without. The most commonly affected domain of quality of life was the psychological domain, regardless of the presence of psychiatric co-morbidities, but patients with psychiatric morbidities were relatively more affected. The least commonly affected domain of quality of life in psoriasis patients was the environmental domain, regardless of the presence of psychiatric morbidities, but patients with psychiatric morbidities were relatively more affected.

**Table 5:** Comparison of quality of life among patients with and without psychiatric morbidities.

Quality of life	With Psychiatric Morbidities(n=55)		Without Psychiatric Morbidities(n=35)		p-value
	Mean	SD	Mean	SD	
Physical	43.36	7.88	52.90	8.69	p<0.001
Psychological	38.12	6.67	48.76	6.21	p<0.001
Social relationship	55.85	9.26	64.43	7.47	p<0.001
Environment	56.67	10.65	64.67	8.18	p<0.001

## Discussion

The majority of the patients in this study were 31-40 years of age, male, married, from urban domiciles, from the upper-lower socioeconomic scale as classified by the Kuppuswamy scale updated for the year 2019, from lower educational backgrounds and belonged to a joint family. The demographics are similar to other studies [11-20].

In this study, 61% of psoriasis patients had psychiatric morbidities, whereas 39% had none. This result was comparable to that of Singh et al. [13] in 2016, Kashyap et al. [13] in 2016, and Paradesi et al. [11] in 2019, who reported psychiatric morbidity rates of 47%, 48%, and 74% in psoriasis patients, respectively. In our study, it was evident that the most common psychiatric morbidity associated with psoriasis was depressive episode (51.51%) followed by anxiety disorder, unspecified (24.24%) and social phobia (9.09%), which was consistent with the existing literature. Muffadel et al. [21] in 2014 and Deshmukh et al. [22] in 2015 also found depressive episode to be the most common psychiatric morbidity in psoriasis patients, followed by anxiety disorder, unspecified.

The prevalence of psychiatric morbidities was found to be highest in the age group of 21-30 years; in other words, psychiatric morbidities were more common in younger age groups, as seen in a 2004 study done by Gelfand et al. [23]. When gender was taken into account, although psoriasis was more prevalent among male patients in this study, psoriasis with psychiatric morbidities was more common in female patients, similar to the study done by Boehm et al. [24]. This result may have been due to a higher concern for external appearance among female patients.

This study also found that although the majority of the psoriasis patients came from urban areas, psychiatric morbidities were comparatively less in these patients compared with those from rural areas, which was comparable to the 2015 study done by Lakshmy et al. [25]. In this study, on evaluating the educational background of the study population, psoriasis with psychiatric morbidities was higher in lower education backgrounds, as comparable with the study done by Chen et al. [26]. Having a rural background and a lower educational level may lead to weaker coping strategies and, thus, more psychiatric morbidities in these patients. These findings can be described by the fact that patients from a rural background usually belong to a lower economic status, have never-ending financial burdens, lower education, difficulties with regard to basic amenities and access to hospitals. Hence, these patients are likely to have poor adherence to treatment and regular follow-ups, which further worsens the severity of psoriasis. It was also evident from this study that psychiatric morbidities in psoriasis patients were more prevalent in the upper lower class as classified by the Kuppuswamy Socioeconomic Scale, as seen in the study done by Paradesi et al. [11]. This study also showed that psychiatric morbidities were more prevalent in psoriasis patients living in a joint family comprising more members as compared to a nuclear family, which was similar to a 2011 study done by Sampogna et al. [27]. One possible reason for this outcome is social stigma among family members. On evaluating marital status, psychiatric morbidities in psoriasis patients were more prevalent in the married population as compared to other marital-status groups, as comparable to the findings of Paradesi et al. [11].

Finally, the results showed that every domain of quality of life was affected in both groups but with a higher intensity in psoriasis patients with psychiatric morbidities as compared to those without.

The most affected domain of quality of life was the psychological domain in both groups, which showed the lowest WHOQOL score, whereas the least affected domain was the environmental domain. Furthermore, if we compare these two specific domains in particular, we also see that patients with psychiatric morbidities had lower scores in both the psychological (38.12 ± 6.67 vs. 48.76 ± 6.21) as well as the environmental (56.67 ± 10.65 vs. 64.67 ± 8.18) domains, as compared with patients without psychiatric morbidities. This result was comparable with previous studies done by Sanyal et al. [28] in 2015 and Mahawer et al. [29] in 2016, in which the most affected domain of quality of life was the psychological domain in psoriasis patients with psychiatric morbidities.

## Conclusions

Our study aimed to assess psychiatric morbidities in psoriasis patients. We found that psychiatric morbidities were a frequent occurrence in psoriasis patients. We also found that more psychiatric morbidities in psoriasis patients led to a poorer quality of life, which further necessitates the need for the early diagnosis of psychiatric morbidities in psoriasis patients so that they do not hamper the quality of life as the disease progresses. Therefore, the collaboration of dermatologists and psychiatrists is critical in controlling psychiatric morbidities in patients with psoriasis at the earliest stage possible.
